# The Development and Testing of a Temporary Small Cold Storage System: Gas-Inflated Membrane Cold Storage

**DOI:** 10.3390/foods15020231

**Published:** 2026-01-08

**Authors:** Lihua Duan, Xiaoyan Zhuo, Jiajia Su, Xiaokun Qiu, Limei Li, Wenhan Li, Yaowen Liu, Xihong Li

**Affiliations:** 1 Tianjin Key Laboratory of Integrated Design and On-Line Monitoring for Light Industry & Food Machinery and Equipment, College of Mechanical Engineering, Tianjin University of Science and Technology, Tianjin 300222, China; 2 State Key Laboratory of Food Nutrition and Safety, College of Food Science and Engineering, Tianjin University of Science and Technology, Tianjin 300457, China

**Keywords:** gas-inflated membrane cold storage (GIMCS), cotton-filled gas-inflated membrane cold storage (CF-GIMCS), conventional cold storage, preservation, temporary storage, small-scale systems, smallholder farming economy, on-farm storage

## Abstract

At present, conventional cold storage facilities in China are poorly suited to on-farm storage demands for agricultural produce, mainly due to their large spatial requirements, complex and labor-intensive installation procedures, limited portability, and insufficient coverage in rural areas. These limitations significantly contribute to post-harvest losses of perishable crops such as cherry tomatoes. To address this challenge, the present study proposes a compact and temporary cold storage system—gas-inflated membrane cold storage (GIMCS)—which exploits the inherent safety, cost-effectiveness, ease of deployment, and adaptability of inflatable membrane structures. A series of mechanical performance tests, including tensile strength, pressure resistance, and burst tests, were conducted on PA/PE (Polyamide/Polyethylene) composite membranes. The optimal configuration was identified as a membrane thickness of 70 μm, a gas column width of 2 cm, and a PA/PE composition ratio of 35%/65%. Thermal performance evaluations further revealed that filling the inflatable structure with 100% CO_2_ yielded the most effective insulation. Through structural optimization, a cotton-filled gas-inflated membrane cold storage system (CF-GIMCS) incorporating a dual insulation strategy—combining intra-membrane and extra-membrane insulation—was developed. This multilayer configuration significantly reduced conductive and convective heat transfer, resulting in enhanced thermal performance. A comparative evaluation between GIMCS and a conventional cold storage system of equivalent capacity was conducted over a 15-day storage period, considering construction cost, temperature uniformity, and fruit preservation quality. The results showed that the construction cost of GIMCS was only 38% of that of conventional cold storage. The internal temperature distribution of GIMCS was highly uniform, with a maximum horizontal temperature difference of 1.4 °C, demonstrating thermal stability comparable to conventional systems. No statistically significant differences were observed between the two systems in key post-harvest quality indicators, including weight loss and respiration rate. Notably, GIMCS exhibited superior performance in maintaining fruit firmness, with a hardness of 1.30 kg·cm^−2^ compared to 1.26 kg·cm^−2^ in conventional storage, indicating a potential advantage in shelf-life extension. Overall, these findings demonstrate that GIMCS represents an affordable, technically robust, and portable cold storage solution capable of delivering preservation performance comparable to—or exceeding—that of conventional cold storage. Its modularity, mobility, and ease of relocation make it particularly well suited to the operational and economic constraints of smallholder farming systems, offering a practical and scalable pathway for improving on-farm cold chain infrastructure.

## 1. Introduction

Cherry tomatoes (*Lycopersicon esculentum* var. *cerasiforme*) are cultivated worldwide and are highly valued for their desirable flavor and rich nutritional profile. They contain abundant proteins, essential minerals, and vitamins, with vitamin C levels reported to be up to 1.7 times higher than those of conventional tomato cultivars [1]. These attributes have driven steadily increasing consumer demand. However, cherry tomatoes are characterized by a high moisture content and a thin cuticle, rendering them highly susceptible to microbial contamination and physiological deterioration during post-harvest handling and storage. Consequently, rapid softening, nutrient degradation, and shortened shelf life frequently occur [2]. In China, agricultural production systems are predominantly fragmented and dominated by small-scale, family-operated farms [3]. Existing cold storage facilities are generally designed for large-scale operations, requiring substantial labor for installation and lacking flexibility in dismantling and relocation. As a result, such facilities are poorly matched to the logistical conditions and financial capacities of smallholder farmers. The limited availability of appropriate cold storage infrastructure further exacerbates post-harvest losses, particularly for highly perishable horticultural products such as cherry tomatoes. This challenge is not unique to China but is widely observed across developing regions. According to the Food and Agriculture Organization of the United Nations, smallholder farms contribute approximately one-third of global food production [4]. In Africa, small-scale farmers manage nearly 80% of cultivated land, yet limited financial resources constrain investment in agricultural infrastructure, leading to severe post-harvest losses [5,6]. Affognon et al. reported that food insecurity affecting millions in eastern and southern sub-Saharan Africa is closely linked to inadequate on-farm storage capacity [7]. There is broad consensus that improving post-harvest technologies represents one of the most effective strategies for reducing food loss and enhancing food system resilience [8]. In China, traditional storage methods include underground cellars [9], naturally ventilated warehouses [10], and modular temperature-controlled units [11]. Although earthen cellars are among the earliest preservation techniques, their unstable internal environments result in high spoilage rates, which led to their gradual abandonment. Naturally ventilated warehouses offer low construction costs and minimal energy consumption but lack mobility and adaptability. Modular cold storage units are easier to assemble and operate; however, their high initial investment costs limit adoption among resource-constrained farmers. Internationally, container-based cold storage systems are commonly deployed in low- and middle-income countries [12], yet their high procurement costs, energy demands, and operational inefficiencies reduce their suitability for decentralized farming contexts. In contrast, developed countries such as the United States, Japan, and the Netherlands have established integrated cold chain systems spanning pre-cooling, storage, refrigerated transport, wholesale distribution, retail display, and household refrigeration. Despite their effectiveness, these systems are technologically complex and financially prohibitive for small-scale agricultural operations.

From a thermodynamic perspective, heat transfer occurs spontaneously from regions of higher temperature to lower temperature through conduction, convection, and radiation [13]. In conventional cold storage systems, heat transfer involves convective exchange between the external environment and the enclosure surface, conductive transfer through the enclosure materials, and internal convection between the enclosure interior and the stored air. Among these mechanisms, conductive heat transfer through the enclosure envelope constitutes a critical determinant of overall thermal resistance, governed by material thermal conductivity, enclosure thickness, and temperature gradients.

Gas-inflated membrane structures have been widely adopted due to their advantages in safety, cost-effectiveness, rapid construction, and design flexibility. Representative applications include temporary structures used during the 2008 U.S. presidential election and gas-inflated membrane sports facilities, such as tennis courts at the Central Party School of the Communist Party of China. More broadly, these structures are increasingly employed in airports, passenger terminals, industrial plants, and large-scale warehouses [14,15,16]. In consumer applications, gas-inflated membranes are commonly used in protective packaging, where trapped air layers provide effective cushioning and insulation [17]. Inspired by this principle, the present study exploits the enclosed gas within inflatable membranes as a passive thermal insulation layer, optimizing the balance among conduction, convection, and radiation to minimize heat ingress. By systematically optimizing the PA/PE composite membrane composition and lamination structure, this study develops a novel dual-insulation cold storage system integrating both intra-membrane and extra-membrane thermal barriers. Termed gas-inflated membrane cold storage (GIMCS), this system combines low material and construction costs with high portability and rapid deployment. As such, it offers a practical, scalable solution for improving post-harvest preservation of perishable crops such as cherry tomatoes and addressing critical cold chain deficiencies in smallholder farming economies.

## 2. Materials and Methods

### 2.1. Materials

#### 2.1.1. Experimental Materials and Reagents

Gas-inflated membrane rolls (standard widths of 90, 100, and 110 cm) were obtained from Cangnan Binqiang Packaging Co., Ltd. (Wenzhou, China). Rigid polyurethane insulation panels (90 cm × 90 cm × 20 cm) used for conventional cold storage were purchased from Shanghai Yejun Refrigeration Equipment Co., Ltd. (Shanghai, China). PVC pipes (3 cm diameter) and corresponding three-way connectors (3.2 cm diameter) were supplied by Tianjin Dongli Xinfangsheng Hardware and Building Materials Branch (Tianjin, China). Multilayer PA/PE composite membranes with nominal thicknesses of 30, 50, 70, and 90 μm were purchased from Tianjin Binhai Building Materials Market (Tianjin, China). A total of 3 kg of Ca-201-PE adhesive was obtained from Dongguan Chuangcheng Adhesive Products Co., Ltd. (Dongguan, China). In addition, 200 polyethylene fresh-keeping bags and 10 kg of cherry tomatoes were purchased from Tianjin Binhai Jinbaorao Wholesale Market (Tianjin, China). Analytical-grade reagents, including carboxymethyl cellulose, galacturonic acid, carbazole, and anhydrous ethanol, were supplied by Tianjin Damo Chemical Reagent Factory (Tianjin, China). A commercial peroxidase (POD) activity assay kit was obtained from Beijing Solarbio Science and Technology Co., Ltd. (Beijing, China).

#### 2.1.2. Experimental Instruments and Equipment

The thermal conductivity tester (VQ-300П) was supplied by Beijing Jujinghong Automation Control Technology Co., Ltd. (Beijing, China). A foot-operated heat sealer (SF-B) and a multifunctional continuous film sealing machine (SF-400) were obtained from Wenzhou Xingye Machinery Equipment Co., Ltd. (Wenzhou, China) and Tianjin Honglian Ruicheng Food Packaging Machinery Co., Ltd. (Tianjin, China), respectively. Mechanical properties were measured using a digital electronic tensile testing machine (TM2101-T5; Guangzhou Luge Precision Instrument Co., Ltd., Guangzhou, China). Temperature monitoring was conducted using a multi-point temperature tester (MIK-R6000F; Hangzhou Meikong Automation Technology Co., Ltd., Hangzhou, China), a digital probe thermometer (11000; DELTATRAK, Pleasanton, CA, USA), and a high-precision multi-channel temperature data logger (F59; Fluke Corporation, Shanghai, China). Additional equipment included an air pump compressor (ZP-KYJ-002; German Zhipu Tools International Group Co., Ltd., Hong Kong, China), a digital vernier caliper (DL91150; Deli Group Co., Ltd., Ningbo, China), a fruit and vegetable respiration rate analyzer (GXH-3051H; Shijiazhuang Shiya Technology Co., Ltd., Shijiazhuang, China), a handheld digital refractometer (PAL-3; Shanghai Zhuoguang Instrument Technology Co., Ltd., Shanghai, China), and a fruit hardness tester (GY-4; Shenzhen Huafeng Technology Co., Ltd., Shenzhen, China). Mass and thermal measurements were performed using electronic balances (AUY120 and JJ-1000; Ohaus Instruments, Changzhou, China, and Mettler-Toledo Instruments, Shanghai, China, respectively) and an electric constant-temperature water bath (HH-2; Ohaus Instruments). Other laboratory equipment included an induction cooker (C22-IJ59E; Supor, Hangzhou, China), a benchtop centrifuge (TG-80; Xiangyi Centrifuge Co., Ltd., Hunan, China), and a microplate spectrophotometer (BioTek Epoch 2; Shanghai Qiqian Electronic Technology Co., Ltd., Shanghai, China).

### 2.2. Experimental Treatments

#### 2.2.1. Development of Gas-Inflated Membrane Cold Storage (GIMCS)

##### Selection of Membrane Thickness

Four types of gas-inflated membrane materials with nominal thicknesses of 30, 50, 70, and 90 μm were selected based on previous research. All membranes had a gas column width of 2 cm and a PA/PE composition ratio of 25%/75%. Tensile strength and pressure resistance tests were conducted to evaluate mechanical performance, and the optimal membrane thickness was determined through comparative analysis.

##### Screening of Gas Column Width

Three gas-inflated membrane materials with a thickness of 70 μm and a PA/PE ratio of 35%/65% were prepared, differing only in gas column width (2, 3, and 4 cm). Thermal resistance measurements were first conducted at the material level. Subsequently, three independent 1 m^3^ cold storage units were fabricated using each membrane type to systematically compare heat conduction performance under different gas column configurations.

##### Screening of PA/PE Composition Ratio

Gas-inflated membranes with a thickness of 70 μm and a gas column width of 2 cm were fabricated using three PA/PE composition ratios: 25%/75%, 30%/70%, and 35%/65%. Static compression resistance and heat-sealing performance tests were conducted, and the optimal PA/PE ratio was identified based on overall mechanical and processing performance.

##### Selection of Insulating Gas

Candidate gases were initially screened based on safety, availability, and cost considerations. Each selected gas was introduced into the gas-inflated membrane, and the resulting thermal conductivity was measured using a thermal conductivity tester. The optimal insulating gas was determined through comparative analysis.

#### 2.2.2. Determination and Optimization of Gas-Inflated Membrane Layers

##### Determination of the Number of Membrane Layers

Carbon dioxide (CO_2_) was introduced into gas-inflated membranes (30 cm × 30 cm, thickness 0.08 mm). The number of membrane layers was increased incrementally, and the corresponding thermal conductivity was measured using a calibrated thermal conductivity tester. The optimal number of layers was determined based on the measured insulation performance.

##### Structural Optimization of Multilayer Membrane Systems

**Figure 1:**
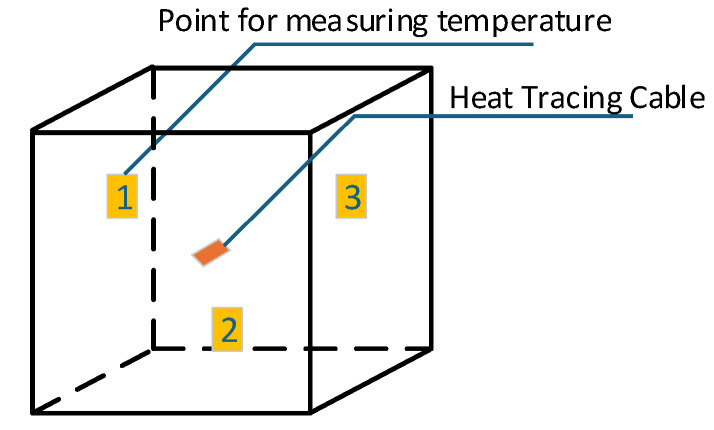
Schematic diagram of temperature measurement using a multi-point thermometer.

(1) Optimization of gas column orientation: Three layers of CO_2_-filled gas-inflated membranes were assembled into two configurations. In the first configuration, gas columns were aligned to allow line-to-surface heat transfer. In the second configuration, the middle layer was rotated by 90° relative to the outer layers, creating orthogonal intersections that restricted heat transfer to point contacts. Both assemblies were sealed in CO_2_-filled cooking bags, and their effective thermal conductivity was measured to assess the influence of gas column orientation on thermal insulation performance.

(2) Enhancement of insulation performance using cotton interlayers: A composite insulation structure was constructed by inserting cotton layers between adjacent CO_2_-filled gas-inflated membranes, forming a cotton-filled gas-inflated membrane cold storage system (CF-GIMCS). This system and a conventional cold storage unit with identical geometry were placed in a temperature-controlled refrigeration chamber. As illustrated in Figure 1, a 2.5 m heating cable was centrally installed and fixed inside each cold storage unit. Three temperature monitoring points (labeled 1, 2, and 3) were positioned at identical locations in both systems. After the internal temperature stabilized at approximately 0 °C, continuous temperature data were recorded using a multi-point temperature measurement system. These data were used to calculate total thermal resistance, enabling a direct comparison of insulation performance under identical boundary conditions.

#### 2.2.3. Performance Testing of GIMCS

**Figure 2:**
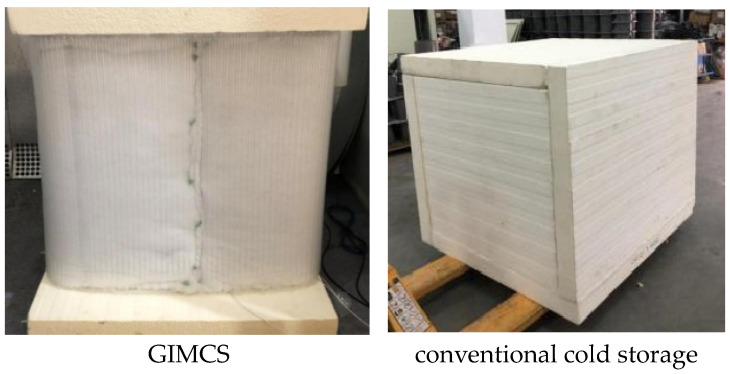
Photographs of the GIMCS and conventional cold storage systems.

Two cold storage units with identical internal volumes—each measuring 5.26 × 2.63 × 3.73 m (approximately 50 m^3^)—were constructed for comparative evaluation. One unit employed the gas-inflated membrane cold storage (GIMCS) system, while the other followed a conventional cold storage design. A schematic representation of both systems is shown in Figure 2.

##### Cost Estimation

Construction costs of the two cold storage systems were calculated and compared based on detailed cost inventories, including material, insulation, and structural components, as summarized in the cold storage construction cost table.

##### Temperature Distribution Testing

**Figure 3:**
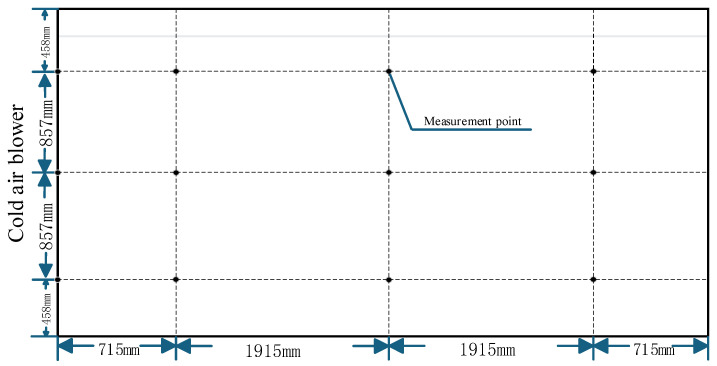
Horizontal layout of temperature measurement points.

To ensure accurate and standardized evaluation of temperature distribution, temperature measurement points were arranged in accordance with cold storage testing specifications. Monitoring locations were divided into three vertical layers: upper, middle, and lower, positioned at heights of 3.7 m, 2.6 m, and 1.3 m above the floor, respectively. Identical horizontal sensor layouts were maintained at each vertical level to enable spatially consistent temperature comparisons. The planar arrangement of the measurement points is illustrated in Figure 3.

##### Evaluation of Cherry Tomato Preservation Performance

Cherry tomatoes were selected based on strict criteria, including uniform size, absence of pests and diseases, no mechanical damage, and full color development, to ensure sample homogeneity. Both the conventional cold storage and GIMCS were operated at a controlled temperature of 5 ± 0.5 °C, with relative humidity maintained at 85–90% under natural convection. Automatic defrosting was conducted at 12 h intervals to prevent frost accumulation and maintain stable operating conditions. The total storage duration was 15 days. Within each cold storage unit, an iron rack was positioned 10 cm above the floor at the central location, supporting a plastic container. Each container held 12 polyethylene (PE) preservation bags, with 20 cherry tomatoes per bag. Four bags per storage system were designated for periodic determination of weight loss and decay rates. At three-day intervals, one bag was randomly selected from each storage system, and 15 fruits were sampled from that bag to form three replicates for quality assessment. Mean values were calculated to enable reliable comparison of preservation performance between the two storage systems.

**Figure 4:**
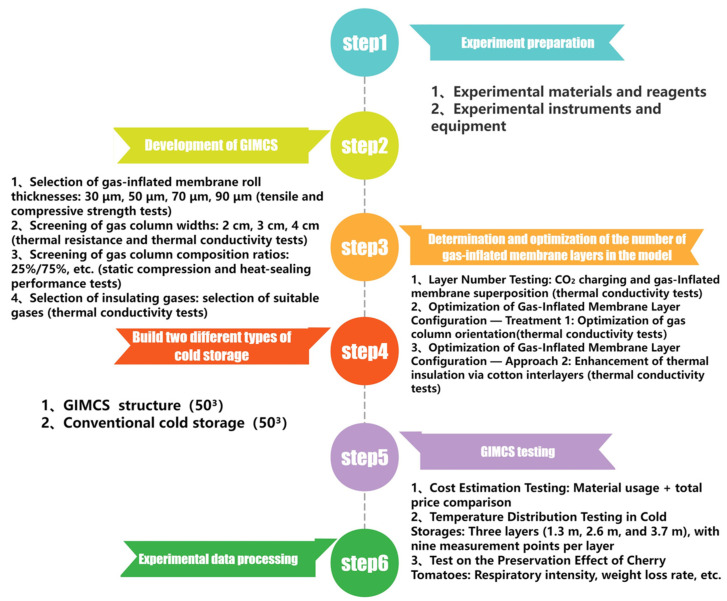
Flowchart of experimental procedures.

A schematic overview of the complete experimental workflow is presented in Figure 4.

### 2.3. Methods

#### 2.3.1. Tensile Strength Testing of Gas-Inflated Membranes

Tensile strength testing was conducted following the procedure described by Li et al. [18]. Test specimens were cut into square samples measuring 20 mm × 20 mm to ensure dimensional consistency. Tensile properties were evaluated in both longitudinal and transverse directions in accordance with GB 13022–91 [19] (Standard Test Method for Tensile Properties of Plastic Membranes). All tests were performed at a constant crosshead speed of 300 mm/min under controlled conditions.

#### 2.3.2. Pressure-Bearing Capacity of Gas Columns

The pressure-bearing capacity of gas columns was evaluated using the method proposed by Ming [20]. During testing, pressure was gradually applied, and the resulting stress–strain behavior was recorded and analyzed to characterize the mechanical response of the gas columns under compressive loading.

#### 2.3.3. Burst Pressure Testing of Gas Columns

For each membrane thickness, six replicate samples were tested to ensure reproducibility. Gas columns were pressurized using a regulated gas supply connected to an electric air pump and a calibrated digital pressure gauge, allowing real-time monitoring of internal pressure. Inflation was continued until rupture occurred. The maximum pressure recorded immediately prior to failure was defined as the burst pressure and used as an indicator of structural integrity.

#### 2.3.4. Thermal Resistance Measurement of Gas-Inflated Membranes

Thermal resistance of the gas-inflated membranes was measured using the guarded hot plate method with a VQ-300П (supplied by Beijing Jujinghong Automation Control Technology Co., Ltd. Beijing, China) thermal conductivity tester following the procedure described in Ref. [21].

#### 2.3.5. Thermal Performance Testing of Cold Storage Enclosures

Three gas-inflated membrane cold storage units were placed inside a large environmental chamber maintained at a constant temperature of 0 °C to simulate a stable external environment. A heating cable of identical length (30 cm) was installed at the center of each cold storage to provide a uniform internal heat source. Six multi-point temperature probes were evenly distributed within each storage unit to monitor internal temperature distribution, while an additional probe was placed outside the cold storage units but within the environmental chamber to record ambient temperature. Once the ambient temperature stabilized at 0 °C, internal temperature data were continuously recorded. The experiment was repeated with increasing numbers of gas-inflated membrane layers, enabling systematic comparison of thermal insulation performance under identical boundary conditions.

#### 2.3.6. Static Compression Testing

Static compression testing was conducted following the method described by Zhang et al. [22]. Compressive stress (σ) was calculated using Equation (1):

(1) $$\sigma =\frac{P}{A}\;\times \;100\%$$

where σ is the compressive stress (Pa), P is the applied compressive load (N), and A is the bearing area of the specimen (mm^2^).

Compressive strain (ε) was calculated using Equation (2):

(2) $$\varepsilon =\frac{T-Tj}{T}$$

where T is the initial thickness of the specimen (mm) and Tj is the thickness after compression (mm).

#### 2.3.7. Heat-Sealing Performance Testing

**Figure 5:**
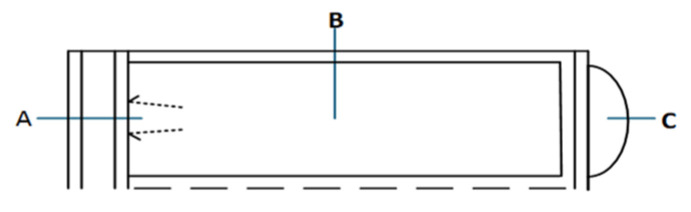
Heat-sealing process for membrane closure. A: the valve region; B: the central section; C: the tail sealing region.

Specimens were collected from three regions of the gas-inflated membrane—the valve region (Area A), central region (Area B), and tail sealing region (Area C)—as shown in Figure 5. Ten specimens were obtained from each region. Each specimen had an unfolded length of 100 ± 1 mm and a width of 15 ± 0.1 mm. Testing followed the procedure described by Zhang et al. [23]. The heat-sealed section was positioned at the center of each specimen, and the specimen arms were opened to an angle of 180° before being clamped into the grips of the tensile testing machine. Care was taken to align the specimen axis with the grip centerline to prevent eccentric loading. The initial grip separation was set to 50 mm, and testing was performed at a crosshead speed of 300 ± 20 mm/min. The maximum load recorded at failure was defined as the heat-sealing strength.

#### 2.3.8. Preliminary Screening of Insulating Gases

Candidate gases were preliminarily screened based on key physicochemical properties, including thermal conductivity and safety considerations. Potential gases were identified through a comprehensive review of relevant literature [24], providing a rational basis for subsequent experimental selection and evaluation.

#### 2.3.9. Calculation of Thermal Conductivity of Gas Mixtures

Selected gases were mixed at different molar ratios, and the thermal conductivity of each gas mixture was calculated using the following theoretical model. Under constant temperature and atmospheric pressure, the thermal conductivity of a gaseous mixture can be estimated as:

(3) λm=∑λiyiMi13∑yiMi13

where

$\lambda_{m}$ is the thermal conductivity of the gas mixture (W·m^−1^·K^−1^);

$\lambda_{i}$ is the thermal conductivity of component *i* (W·m^−1^·K^−1^);

$y_{i}$ is the molar fraction of component *i* in the mixture;

$M_{i}$ is the molar mass of component *i* (kg·kmol^−1^).

#### 2.3.10. Measurement of Thermal Conductivity After Membrane Inflation

Thermal conductivity measurements were performed using a calibrated thermal conductivity tester to ensure accuracy and repeatability. Individual gases were introduced into a gas-inflated membrane with a fixed internal volume of 30 × 30 × 2 cm^3^. The inflated membrane was placed between a hot plate and a cold plate to establish a stable one-dimensional heat transfer pathway. Prior to measurement, the sample was gently compressed by the testing apparatus to eliminate interfacial air gaps. Measurements were conducted under steady-state conditions for 180 min to ensure complete thermal equilibrium. During testing, the cold plate temperature was maintained at 0 °C, while the hot plate was held at 50 °C, thereby establishing a constant and reproducible temperature gradient across the specimen.

#### 2.3.11. Cost Estimation of Cold Storage Systems

During cold storage construction, the quantities and corresponding costs of insulation materials and auxiliary components required to achieve equivalent thermal performance were systematically recorded. Total construction costs were calculated for each cold storage configuration, enabling a quantitative comparison of cost efficiency among different systems and supporting objective evaluation of economic feasibility.

#### 2.3.12. Temperature Distribution Testing Within the Cold Storage

Temperature probes equipped with wireless transmission modules were securely fixed at predefined measurement locations inside the cold storage using rubber bands or transparent adhesive tape. Each probe location was clearly labeled to ensure traceability. Temperature acquisition commenced 25 min after the cooling system was activated, allowing sufficient time for thermal stabilization. Collected temperature data were imported into Origin software 2021 for structured processing and analysis, enabling systematic comparison of temperature distribution across different spatial locations within the cold storage.

#### 2.3.13. Evaluation of Cherry Tomato Preservation Quality

The respiration rate, weight loss, firmness, and total soluble solids (TSS) content of cherry tomatoes were determined following the standardized methods described by Du Junmei [25]. Cellulase (Cx) and pectin methylesterase (PME) activities were measured according to the protocol reported by Lohani et al. [26]. Pectin content was quantified by integrating the methods described by Xie et al. [27], Qian Chunlu et al. [28], and Liu Chuanben et al. [29], thereby improving analytical reliability. Superoxide dismutase (SOD) and peroxidase (POD) activities were analyzed following the method of Cao Jiankang [30]. Catalase (CAT) activity was determined using a commercial assay kit in strict accordance with the manufacturer’s instructions to ensure consistency and accuracy across samples.

### 2.4. Experimental Data Processing

Unless otherwise stated (Section 2.3.3), all physical measurements were performed with three technical replicates, and all physiological and biochemical assays were conducted with three biological replicates. Experimental data were processed using Excel 2022 and are presented as mean ± standard deviation. Post hoc multiple comparisons were performed using Duncan’s multiple range test, with statistical significance defined at *p* < 0.05. All statistical analyses and graphical visualizations were conducted using Origin software 2021.

## 3. Results

### 3.1. Development of Gas-Inflated Membrane Cold Storage

#### 3.1.1. Selection of Materials for Gas-Inflated Membrane Structures

A wide variety of gas-inflated membrane materials is commercially available, offering different balances between mechanical performance and thermal stability. In the present study, membrane thicknesses ranged from 30 to 90 μm. The width of individual gas columns was 2–8 cm in the uninflated state and expanded to approximately 2–4 mm after inflation. The investigated PA/PE composition ratios were 25%/75%, 30%/70%, and 35%/65%, which are known to exert a significant influence on the physicochemical properties of the membrane. An increased PA content enhances mechanical strength, rigidity, and resistance to elevated temperatures but reduces flexibility and elongation. Conversely, higher PE content improves ductility and tensile extensibility at the expense of stiffness, strength, and thermal resistance [31]. Given these trade-offs, identifying an optimal membrane configuration is critical for achieving reliable mechanical performance while maintaining cost-effectiveness in cold storage applications. Therefore, membrane materials were systematically evaluated based on tensile properties, pressure-bearing capacity, and burst pressure behavior.

##### Selection of Gas-Inflated Membrane Thickness

(1) Tensile properties

As shown in Table 1, gas-inflated membranes of identical thickness exhibited slightly higher tensile strength in the longitudinal direction than in the transverse direction, indicating moderate mechanical anisotropy. Significant differences in tensile strength were observed among membranes of different thicknesses. Both longitudinal and transverse tensile strengths increased with membrane thickness up to 70 μm, reaching peak values of 35.73 ± 0.03 N/mm^2^ and 34.92 ± 0.02 N/mm^2^, respectively. Further increases in thickness resulted in a decline in tensile strength. This non-linear trend suggests that a thickness of 70 μm represents an optimal balance between material continuity and mechanical integrity.

(2) Pressure-bearing capacity

**Figure 6:**
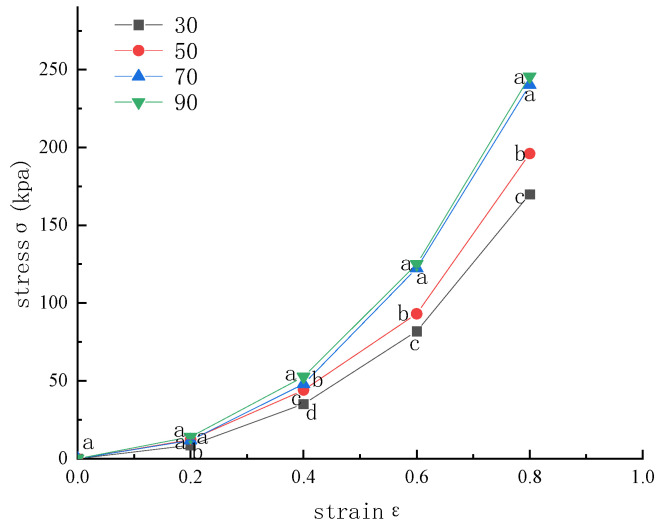
Pressure-bearing capacity of gas-inflated membranes with different thicknesses. Different lowercase letters indicate significant differences among data of different treatment groups (*p* < 0.05).

Stress–strain curves obtained from quasi-static compression tests are presented in Figure 6. The results demonstrate a clear positive correlation between membrane thickness and pressure-bearing capacity. Thicker membranes exhibited enhanced resistance to compressive loading, attributable to improved structural continuity and reduced stress concentration within the gas columns. However, when membrane thickness exceeded 70 μm, further increases produced only marginal improvements in load-bearing performance. Considering both mechanical efficiency and material cost, the 70 μm membrane offers the most favorable compromise for practical application.

(3) Burst pressure behavior

**Figure 7:**
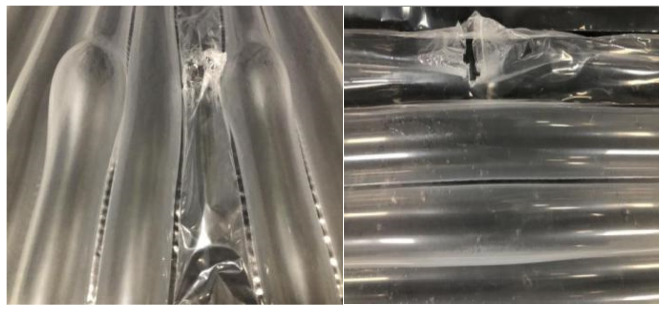
Burst pressure behavior of a single gas column.

Burst pressure test results are summarized in Table 2 and illustrated in Figure 7. Within the thickness range of 30–70 μm, both gas column deformation and burst pressure increased substantially with increasing membrane thickness, indicating enhanced resistance to rupture under internal pressure. Beyond 70 μm, the rate of improvement diminished markedly, suggesting a saturation effect in structural reinforcement. When both safety performance and economic feasibility are considered, the 70 μm membrane thickness emerges as the most rational choice for gas-inflated membrane cold storage systems.

##### Screening of Gas Column Widths

(1) Thermal conductivity

**Figure 8:**
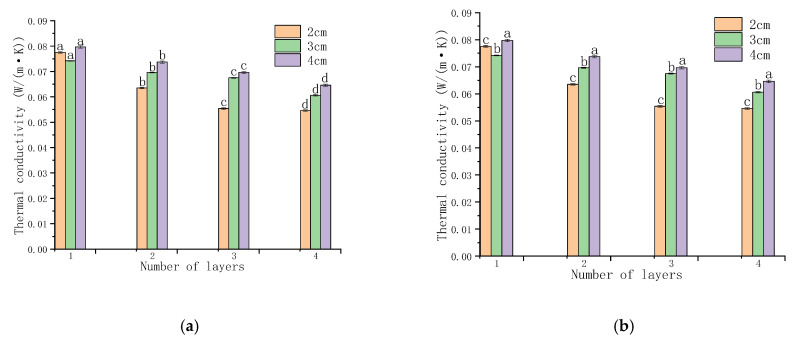
Effect of the number of layers and gas column width on thermal conductivity. (**a**) Comparison by layer count; (**b**) Comparison by column width. Different lowercase letters indicate significant differences among data of different treatment groups (*p* < 0.05).

The experimental results shown in Figure 8 indicate that both gas column width and the number of membrane layers significantly affect thermal conductivity. Although the 3 cm gas column exhibits the lowest initial thermal conductivity, the 2 cm configuration shows the greatest improvement as the number of layers increases. Specifically, its thermal conductivity decreases from approximately 0.08 to 0.05 W/(m·K), reaching the lowest value among all tested configurations at the fourth layer. This trend suggests that wider gas columns facilitate enhanced interlayer convective heat transfer as the number of layers increases, which progressively degrades insulation performance. In contrast, the compact geometry of the 2 cm gas column effectively suppresses convective circulation, thereby maintaining superior thermal resistance. Overall, these results demonstrate that a gas column width of 2 cm provides the most favorable balance between structural design and thermal insulation performance for cold storage applications.

(2) Thermal conductivity of the cold storage enclosure

**Figure 9:**
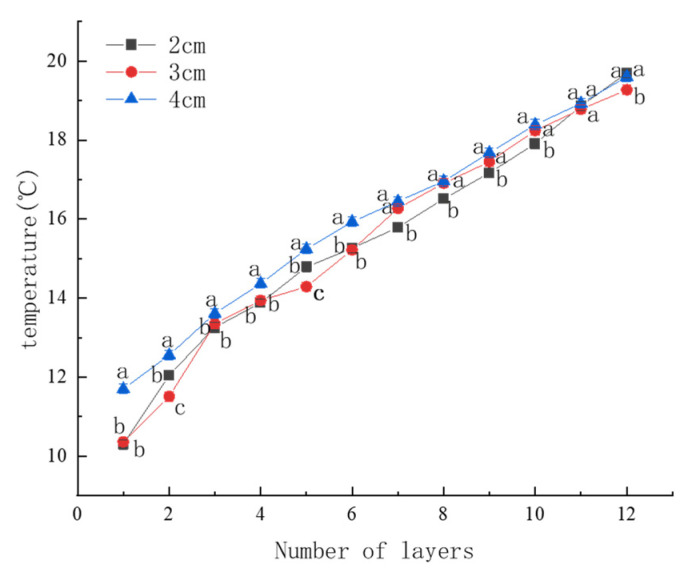
Overall thermal conductivity of membranes with different gas column widths. Different lowercase letters indicate significant differences among data of different treatment groups (*p* < 0.05).

As illustrated in Figure 9, the overall thermal conductivity of the cold storage enclosure varies markedly with both gas column width and layer number. In the low-layer range (1–7 layers), the 3 cm and particularly the 4 cm configurations exhibit lower thermal conductivity than the 2 cm width, indicating an initial insulation advantage. However, this advantage diminishes as the number of layers increases. Between 7 and 11 layers, the differences among the three configurations gradually narrow, and by the 11th layer, all widths achieve nearly identical thermal conductivity values. At the 12th layer, the 2 cm and 4 cm configurations outperform the 3 cm width, with comparable insulation efficiency between the former two. This shift in performance ranking highlights the long-term effectiveness of the 2 cm gas column design, which benefits from enhanced structural continuity and reduced air movement between adjacent columns. Moreover, the consistent decrease in thermal conductivity with increasing layer count confirms that multilayer architectures significantly enhance insulation performance. Collectively, these findings support the selection of a 2 cm gas column width as the optimal configuration for cold storage enclosures, particularly in high-layer systems where convection suppression and thermal stability are critical.

##### Screening of Gas Column Composition Ratios

(1) Pressure-bearing capacity

**Figure 10:**
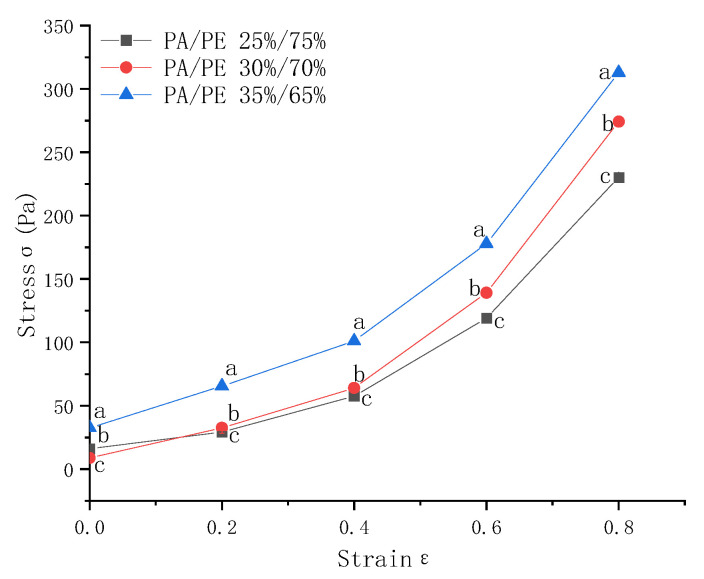
Effect of gas column composition on pressure-bearing capacity. Different lowercase letters indicate significant differences among data of different treatment groups (*p* < 0.05).

The pressure-bearing capacity of gas columns with different PA/PE composition ratios is presented in Figure 10. Clear differences are observed among the tested formulations. The membrane with a PA/PE ratio of 35%/65% exhibits the highest pressure-bearing capacity, demonstrating superior resistance to compressive loading. This behavior is consistent with the known role of polyamide (PA) in enhancing mechanical properties: increased PA content leads to higher chain stiffness and stronger intermolecular interactions, thereby improving structural rigidity and resistance to deformation. Because cold storage enclosures are subjected to repeated mechanical stresses during installation and operation, membranes with a higher PA content—particularly those with a 35%/65% PA/PE ratio—are better suited to maintaining long-term structural integrity. Consequently, this composition offers a clear advantage for practical implementation in gas-inflated membrane cold storage (GIMCS) systems.

(2) Heat-sealing strength

The heat-sealing strength results summarized in Table 3 show that sealing strength is comparable between parts B and C across all composition ratios, reflecting the similarity of their two-layer membrane structures. In contrast, part A exhibits significantly higher sealing strength due to its four-layer configuration, which consists of two outer structural membranes and a two-layer valve membrane. This multilayer architecture increases the effective bonding area and enhances interfacial adhesion. In addition, sealing performance improves consistently with increasing PA content. Membranes with higher PA proportions exhibit stronger seals, higher peel strength, and improved resistance to delamination. This enhancement can be attributed to the superior thermal stability of PA and the stronger molecular entanglement formed within PA-rich regions during the heat-sealing process. As a result, the 35%/65% PA/PE formulation not only achieves optimal sealing integrity but also ensures durability under repeated thermal cycling and mechanical loading. When pressure-bearing capacity and heat-sealing performance are considered together, the 35%/65% PA/PE ratio emerges as the most reliable and technically sound choice for high-performance GIMCS fabrication.

Based on the comprehensive experimental evaluation, a gas-inflated membrane thickness of 70 μm, a gas column width of 2 cm, and a PA/PE composition ratio of 35%/65% are identified as the optimal configuration for insulating materials in GIMCS. From an economic perspective, this selection is strongly supported by previous studies demonstrating the exceptionally low manufacturing cost of polyethylene-based materials [32], enabling high-performance insulation at reduced expense. The choice of a 2 cm gas column width is further corroborated by Yu et al. [33], who reported that porous media with numerous small pores effectively reduce thermal conductivity by suppressing conductive and convective heat transfer pathways. Additionally, Sun et al. [34] demonstrated that polymer blends with higher PA content exhibit enhanced mechanical strength and thermal stability under cryogenic conditions, directly supporting the selected composition ratio. Extensive literature confirms that polymer membranes possess excellent thermal and electrical insulation properties, as well as strong resistance to environmental degradation [35]. In particular, polyethylene-based porous substrates exhibit inherent hydrophobicity, which enhances moisture resistance and ensures long-term stability in high-humidity environments [36]. The suitability of polyethylene for engineering applications is further validated by its well-established mechanical durability and chemical resistance [37]. Moreover, empirical studies have shown that such membranes exhibit high tensile strength and can withstand substantial mechanical stress without rupture, ensuring structural integrity throughout installation and service life [38]. Collectively, these results provide robust, multi-source validation of the proposed material system, confirming its technical feasibility, functional superiority, and practical applicability in real-world cold storage infrastructure.

#### 3.1.2. Selection of Insulating Gases

The gas-inflated membrane cold storage (GIMCS) system consists primarily of the membrane structure and the gas used to inflate it. In the absence of convective heat transfer, gases generally exhibit low thermal conductivity and therefore function effectively as thermal insulators [13]. Once the membrane is inflated, the enclosed gas forms a stable insulating layer that substantially enhances the overall thermal performance of the system. The selection of an appropriate insulating gas requires a systematic and multistep evaluation. Initially, candidate gases are screened based on their fundamental thermophysical properties. Subsequently, binary or multicomponent gas mixtures are comparatively assessed. Key parameters considered in this process include thermal conductivity, cooling performance, chemical stability, safety, and economic feasibility. Through comprehensive analysis of these factors, gas compositions that provide optimal insulation efficiency while remaining cost-effective and operationally safe can be identified, which is essential for the sustainable application of GIMCS.

##### Selection of an Appropriate Gas

(1) Preliminary screening of individual gases

Based on the data presented in Table 4, six gases with relatively low thermal conductivity were initially identified: carbon dioxide, propane, ethylene, argon, ethane, and nitrogen. However, several of these gases present significant safety concerns. Propane is highly flammable, ethylene may induce central nervous system depression at elevated concentrations, and ethane has been reported to cause adverse health effects when concentrations exceed 6%. These risks considerably limit their suitability for practical insulation applications. In contrast, carbon dioxide, argon, and nitrogen are non-flammable, exhibit low physiological toxicity under controlled conditions, and have been widely used in industrial and engineering applications. From the perspectives of operational safety, reliability, and feasibility, these three gases were therefore selected for further analysis, and subsequent evaluations were restricted accordingly.

(2) Calculation and screening of mixed-gas thermal conductivity

Using Equation (3), the thermal conductivity values of mixed gases were calculated, and the results are summarized in Table 5. The data demonstrate a clear decreasing trend in thermal conductivity with increasing carbon dioxide content, indicating a strong dependence on gas composition. The lowest thermal conductivity among the tested mixtures (0.01392 W·m^−1^·K^−1^) was observed for a composition of 90% CO_2_, 5% Ar, and 5% N_2_. However, this value remains slightly higher than that of pure carbon dioxide, which exhibits an even lower thermal conductivity of 0.0137 W·m^−1^·K^−1^. This result highlights the superior insulation performance of pure CO_2_ compared with mixed-gas formulations. Accordingly, pure carbon dioxide was selected as the optimal filling gas for gas-inflated membrane applications.

##### Influence of Gas Composition on the Insulation Performance of Gas-Inflated Membranes

**Figure 11:**
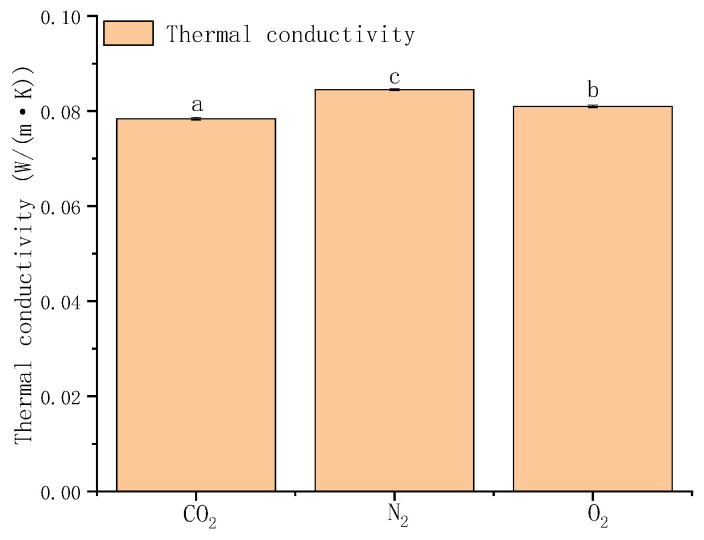
Thermal conductivity of gas-inflated membranes filled with different gases. Different lowercase letters indicate significant differences among data of different treatment groups (*p* < 0.05).

Based on the preliminary screening results, pure CO_2_ was identified as the most promising insulating gas. To further validate its performance, CO_2_, N_2_, and O_2_ were selected as representative gases for comparative experimental evaluation. As shown in Figure 11, the thermal conductivity of gas-inflated membranes varies significantly with the type of gas used. Among the tested gases, membranes filled with pure CO_2_ exhibit the lowest thermal conductivity. For a single-layer membrane with dimensions of 30 × 30 cm, the measured thermal conductivity reaches 0.078375 W·m^−1^·K^−1^, confirming the superior insulating capability of carbon dioxide. These findings are consistent with previous studies demonstrating the excellent thermochemical stability of CO_2_ under typical operating conditions [39]. Furthermore, Han [40] reported that the low thermal conductivity of CO_2_ has been repeatedly verified across multiple independent investigations, lending strong support to the reliability and robustness of the present results.

### 3.2. Determination and Optimization of the Physical Model of GIMCS

With the gas-inflated membrane material and the optimal filling gas identified, the focus shifts to evaluating the feasibility of constructing a functional cold storage system based on this technology. A key engineering question is how many layers of gas-inflated membranes are required to achieve thermal insulation performance comparable to that of a conventional cold storage facility. Addressing this question is essential for assessing the scalability, structural design, and practical applicability of GIMCS, and it is systematically investigated in the following section.

#### 3.2.1. Determination and Optimization of the Number of Gas-Inflated Membrane Layers in the Model

##### Relationship Between the Number of Gas-Inflated Membrane Layers and Insulation Performance

**Figure 12:**
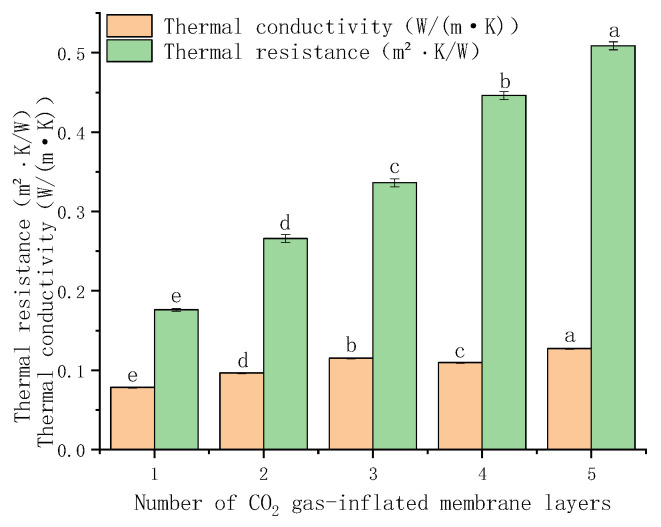
Relationship between the number of optimal CO_2_ gas-inflated membrane layers and thermal conductivity. Different lowercase letters indicate significant differences among data of different treatment groups (*p* < 0.05).

**Figure 13:**
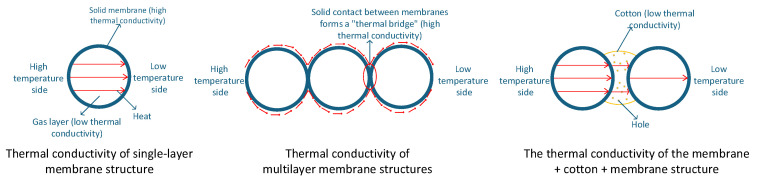
Schematic diagram of heat transfer mechanisms in membranes with different structural configurations.

As shown in the experimental results (Figure 12), the effective thermal conductivity of the gas-inflated membrane material increases from 0.078375 to 0.127171 W·m^−1^·K^−1^ as the number of membrane layers increases. This trend appears counterintuitive, as multilayer structures are generally expected to enhance thermal insulation. The underlying mechanism can be explained by the heat transfer characteristics of gas-inflated membrane systems. In a single-layer gas-inflated membrane, heat transfer occurs primarily through two theoretical pathways: (i) longitudinal conduction along the solid polymer matrix and (ii) transverse conduction across the encapsulated gas cavity between the two membrane surfaces. Under standard conditions, the thermal conductivity of solids is significantly higher than that of liquids and, especially, that of gases—particularly stagnant gases [41]. Although solid-phase conduction is inherently more efficient, the continuous structure of the membrane and the near-uniform in-plane temperature distribution result in an extremely small longitudinal temperature gradient. Consequently, lateral heat flux is minimal, and through-plane heat transfer across the enclosed gas layer becomes the dominant mechanism. Because stagnant gas exhibits exceptionally high thermal resistance, single-layer gas-inflated membranes demonstrate excellent intrinsic insulation performance. In contrast, when multiple gas-inflated membrane layers are stacked and compressed between hot and cold plates during thermal characterization, interstitial air between adjacent layers is progressively expelled by mechanical pressure. This compression suppresses interlayer convection but simultaneously increases the effective contact area between adjacent membrane surfaces. Whether occurring via line–surface or point–point contacts, these localized solid–solid interfaces form continuous thermal bridges that bypass the insulating gas cavities. As a result, heat is no longer required to traverse the high-resistance gas layer; instead, it is rapidly transmitted across successive layers through direct solid conduction. Accordingly, the dominant heat transfer pathway transitions from a “membrane–gas–membrane” configuration in single-layer systems to a “membrane–solid contact–membrane” configuration in multilayer assemblies, as schematically illustrated in Figure 13. This shift leads to a pronounced reduction in interfacial thermal resistance, a marked increase in effective thermal conductivity, and a consequent degradation of insulation performance at the material level. These findings highlight the necessity of refining the physical model of GIMCS by explicitly accounting for the impact of layer stacking and interfacial contact on thermal transport, thereby enabling more accurate prediction and optimization of system performance under realistic operating conditions.

Importantly, these results are fully consistent with the trends observed in Figure 9, which evaluates the thermal insulation performance of gas columns with different widths under practical cold storage conditions. In real systems, overall insulation performance is governed by the combined effects of conduction and convection. The superior insulation performance of multilayer gas-inflated membrane cold storages arises primarily from the compact geometry of the 2 cm gas columns, which effectively suppress convective heat transfer compared with wider columns. This suppression effect becomes more pronounced as the number of layers increases, compensating for the increase in intrinsic material-level thermal conductivity observed in Figure 12. Consequently, despite the higher measured conductivity of stacked membrane materials, the system-level thermal performance improves. These two analyses therefore describe the same phenomenon from complementary perspectives—material-level heat transfer versus system-level structural behavior—and together provide a comprehensive understanding of the insulation mechanism.

##### Optimization of the Number of Gas-Inflated Membrane Layers in the Model

Two distinct strategies were investigated to optimize the number of gas-inflated membrane layers: (i) optimization of gas column orientation and (ii) enhancement of insulation through the introduction of cotton interlayers.

**Figure 14:**
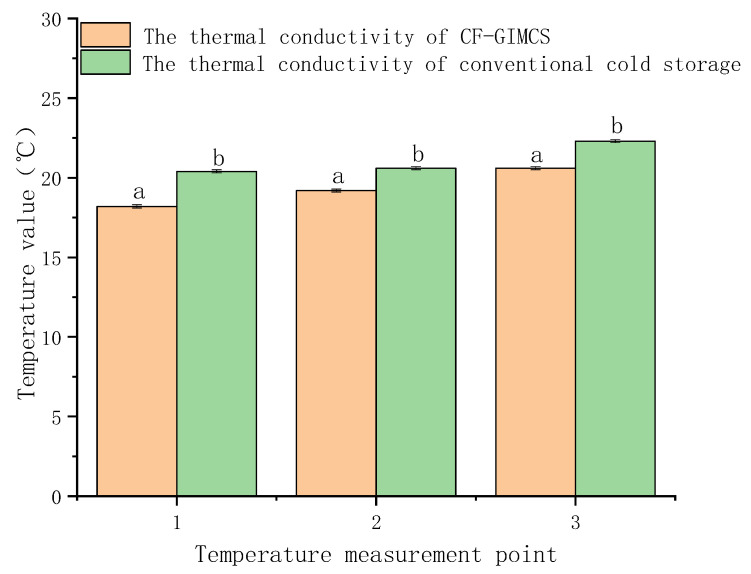
Thermal conductivity of CF-GIMCS and conventional cold storage. Different lowercase letters indicate significant differences among data of different treatment groups (*p* < 0.05).

(1) The effect of gas column orientation on reducing thermal loss is summarized in Table 6. The results indicate that reorienting the gas columns does not lead to a measurable reduction in heat loss, suggesting that orientation alone is insufficient to overcome the formation of solid thermal bridges in multilayer membrane assemblies.

(2) Figure 14 illustrates the performance of a four-layer structure consisting of “gas-inflated membrane + cotton + gas-inflated membrane + gas-inflated membrane”. This configuration exhibits significantly enhanced thermal insulation compared with conventional cold storage systems. The optimized cotton-filled gas-inflated membrane cold storage (CF-GIMCS) achieves a total thermal resistance of 10.9 m^2^·K·W^−1^, substantially exceeding that of standard designs. This level of performance underscores both the technical superiority and the strong commercial potential of CF-GIMCS.

As shown in Figure 13, the incorporation of a cotton interlayer markedly reduces the effective thermal conductivity of the PA/PE composite membrane system. By encapsulating cotton between membrane layers, direct solid–solid contact is effectively eliminated, thereby suppressing conductive heat transfer pathways. Cotton possesses a porous microstructure with a nominal thermal conductivity of approximately 0.023 W·m^−1^·K^−1^, and its interconnected pore network traps a large volume of air. In addition, the hydrophobic nature of the gas-inflated membrane prevents moisture absorption, ensuring that low thermal conductivity is maintained under practical operating conditions. The entrapped air remains largely static, which is critical for minimizing convective heat transfer, as stagnant air exhibits substantially lower thermal conductivity than solid materials. Consequently, unlike conventional multilayer membrane assemblies, CF-GIMCS achieves enhanced insulation through a dual mechanism operating both within and between membrane layers, creating multiple low-conductivity barriers. In all subsequent discussions, the term “GIMCS” refers specifically to this optimized CF-GIMCS configuration. The selection of the four-layer structure is primarily driven by mechanical integrity requirements, which are beyond the scope of the present study.

Previous studies further support these findings. Frydrych et al. [42] systematically evaluated the thermal insulation properties of cotton and Tencel fabrics and demonstrated the superior insulating performance of cotton under comparable conditions. Building on this work, Zayed et al. [43,44] further enhanced the thermal insulation efficiency of cotton fabrics through targeted technical modifications. Collectively, these studies provide strong external validation for the experimental results reported here, confirming the reliability and scientific robustness of the proposed CF-GIMCS design.

#### 3.2.2. Structural Design of the Gas-Inflated Membrane Cold Storage Model and Solutions for Door and Joint Sealing

**Figure 15:**
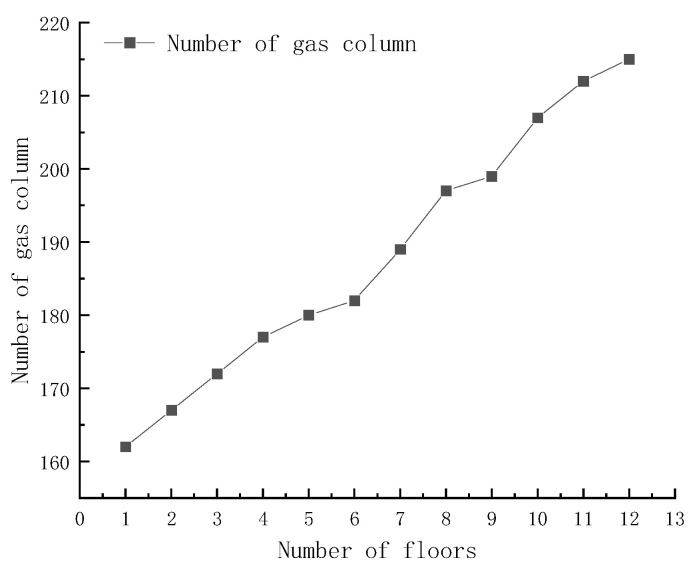
Relationship between the number of membrane layers and gas column number in GIMCS.

In addition to conventional operational subsystems—such as refrigeration, ventilation, and lighting—typically incorporated in standard cold storage facilities, the gas-inflated membrane cold storage system integrates load-bearing walls, a roof, flooring, and doors into a fully self-supporting structural framework. The walls consist of four layers of gas-inflated membranes with an intermediate cotton insulation layer, forming a robust composite structure capable of resisting both internal pressure and external environmental loads. The gas columns are oriented vertically to ensure uniform stress distribution, and adjacent membrane layers are bonded using Ca-201-PE adhesive. A key design parameter is the relationship between the number and dimensions of membrane components at corner joints and the total number of membrane layers, as illustrated in Figure 15. With each additional layer, the number of corner gas columns increases by 2–8, corresponding to a lateral expansion of approximately 20–160 cm per layer. Minor dimensional deviations may arise during assembly due to variations in adhesive bonding strength; however, these deviations remain within acceptable tolerances for modular construction. The flooring system is engineered to provide thermal insulation, seismic resistance, anti-slip performance, and surface flatness. It comprises multiple functional layers, including the sub-base, base layer, insulation layer, vapor barrier, and wearing surface. The roof adopts a flat configuration composed of four membrane layers, with the inner two layers positioned above the outer layers. This arrangement contrasts with the wall structure, where the inner layers are placed beneath the outer ones. Such staggered layering improves sealing performance by minimizing interlayer gaps and enables rapid disassembly and relocation without compromising structural integrity. The cold storage door employs a sliding mechanism and adopts the same sealing principle as the roof, ensuring consistent air tightness and thermal insulation at all interfaces. Unlike conventional steel-framed cold storage units, the GIMCS does not require external supporting structures and functions as a fully self-supporting enclosure. This design substantially reduces construction complexity and dismantling time. As long as the membrane structure remains intact, the facility can be efficiently disassembled, transported, and reassembled for repeated use, supporting circular economy principles. Furthermore, the footprint and geometric configuration of the cold storage can be flexibly adjusted to meet site-specific requirements. Compared with small-scale refrigeration units reported in previous studies [45,46,47,48,49,50], the gas-inflated membrane cold storage demonstrates superior scalability and adaptability. These characteristics make it particularly suitable for decentralized deployment at agricultural production sites and well aligned with the rural infrastructure and supply-chain conditions prevalent in China.

### 3.3. Performance Testing of Gas-Inflated Membrane Cold Storage

#### 3.3.1. Cost Estimation

Polyurethane insulation panels with a thickness of 100 mm typically cost between USD 27.84 and USD 41.76 per square meter and are widely used in conventional cold storage construction despite their relatively high expense. In contrast, the gas-inflated membrane material costs approximately USD 0.42 per square meter, while cotton insulation costs about USD 2.78 per square meter—both substantially lower than traditional rigid foam insulation materials. As summarized in Table 7, the total insulation cost of the GIMCS system is approximately USD 1113.6, whereas the construction cost of a comparable conventional cold storage facility is about USD 3062.4. This corresponds to a cost reduction exceeding 63%, highlighting the pronounced economic advantage of the proposed design. Importantly, this reduction in cost is achieved without compromising thermal performance, as the composite membrane–cotton structure maintains effective thermal resistance.

During construction, it is essential that the gas-inflated membrane structure be fully inflated prior to bonding. Owing to the elastic characteristics of the membrane materials, bonding before inflation would compromise structural integrity and functional performance. The elasticity of the pressurized gas columns is critical for achieving uniform adhesion and maintaining long-term dimensional stability. Overall, the total cost of a 50 m^3^ gas-inflated membrane cold storage facility is approximately 38% of that of a conventional cold storage unit. Compared with phase change material (PCM)-based cold storage systems reviewed by Shoeibi et al. [51,52,53,54,55,56,57,58,59,60] and traditional cold storage solutions, the cotton-filled gas-inflated membrane system exhibits superior cost-efficiency and a simpler structural configuration, significantly enhancing its feasibility for large-scale implementation and commercialization.

#### 3.3.2. Temperature Distribution Within Cold Storages

**Figure 16:**
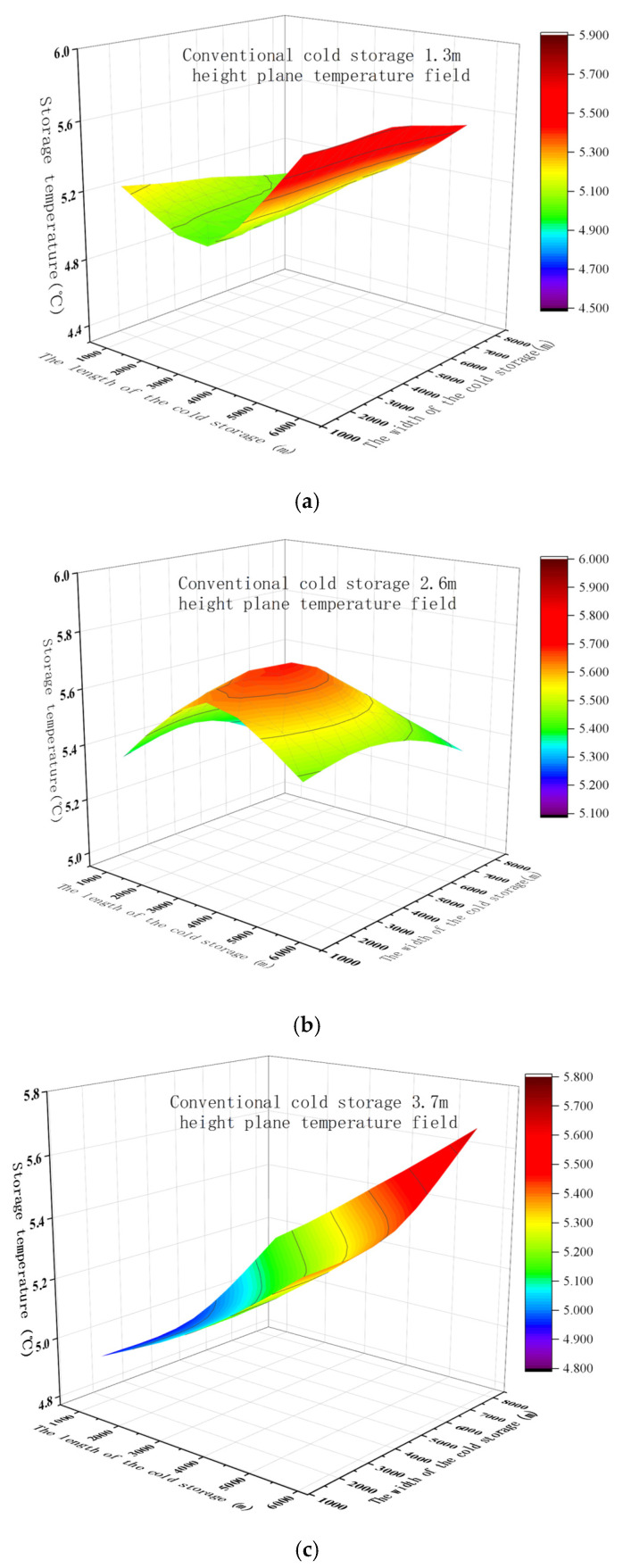

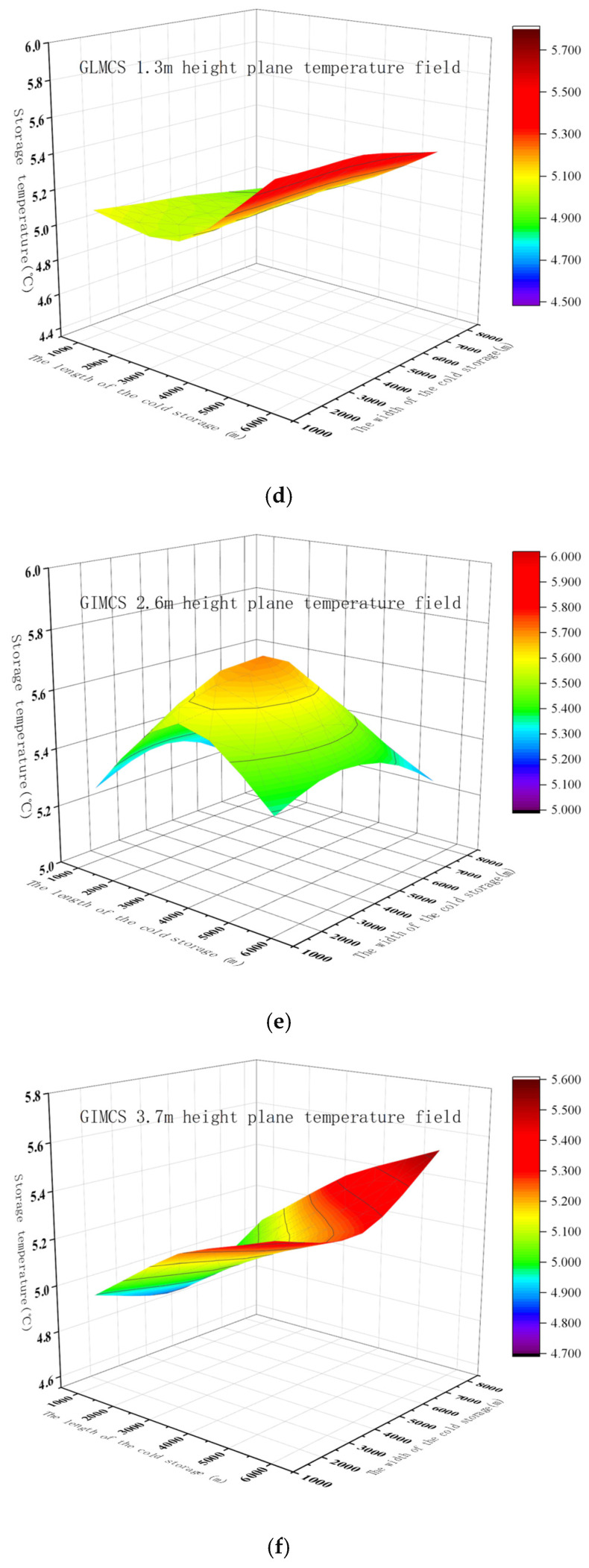
Comparison of temperature distribution within conventional cold storage and GIMCS at various heights: (**a**–**c**) conventional cold storage at (**a**) 1.3 m, (**b**) 2.6 m, and (**c**) 3.7 m; (**d**–**f**) GIMCS at (**d**) 1.3 m, (**e**) 2.6 m, and (**f**) 3.7 m.

The temperature distribution results are presented in Figure 16. A clear spatial temperature gradient is observed, with lower temperatures near the cold air outlet and progressively higher temperatures toward the distal end of the storage chamber. A consistent vertical stratification is also evident, with temperature decreasing as height increases. Minor temperature asymmetries between the left and right walls near the air outlet are likely caused by airflow deflection during propagation or by imperfect sealing at the access door, both of which may disrupt uniform air distribution. Localized temperature elevations at specific interior measurement points are primarily attributed to large-scale vortices formed where the primary airflow impinges on internal surfaces. In these recirculation zones, reduced air velocity limits convective heat transfer, resulting in delayed heat dissipation and localized heat accumulation. For the conventional cold storage (Figure 16a–c), the maximum horizontal temperature difference at the same height is 1.4 °C, while the maximum vertical temperature difference at a single location is 0.6 °C. In the GIMCS (Figure 16d–f), the horizontal temperature variation remains at 1.4 °C, and the vertical difference increases only slightly to 0.7 °C. These results indicate that the temperature uniformity within the membrane-based cold storage is comparable to that of the conventional system. Notably, temperatures at wall surfaces and corner regions are consistently lower in the GIMCS. This improvement is attributed to the arc-shaped enclosure geometry, which eliminates sharp corners and reduces the formation of stagnant air zones, thereby promoting more homogeneous airflow. When combined with the superior thermal insulation properties of the membrane–cotton composite, this geometric advantage contributes to improved microclimate control within the storage chamber.

Compared with previous studies by Zargar [61] and Budiyanto et al. [62], the present results differ in absolute temperature distributions due to variations in chamber design, insulation materials, refrigeration equipment layout, and airflow organization. Nevertheless, the findings consistently demonstrate that the GIMCS achieves thermal performance comparable to, and in some conditions superior to, that of conventional cold storage systems.

#### 3.3.3. Evaluation of the Preservation Effect on Cherry Tomatoes

**Figure 17:**
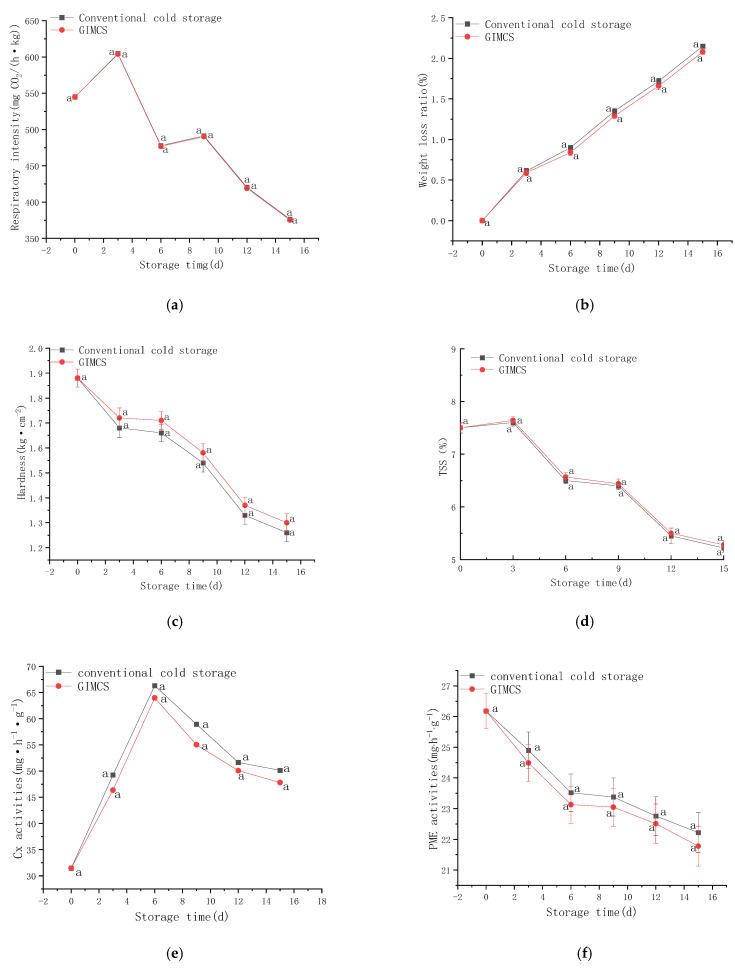

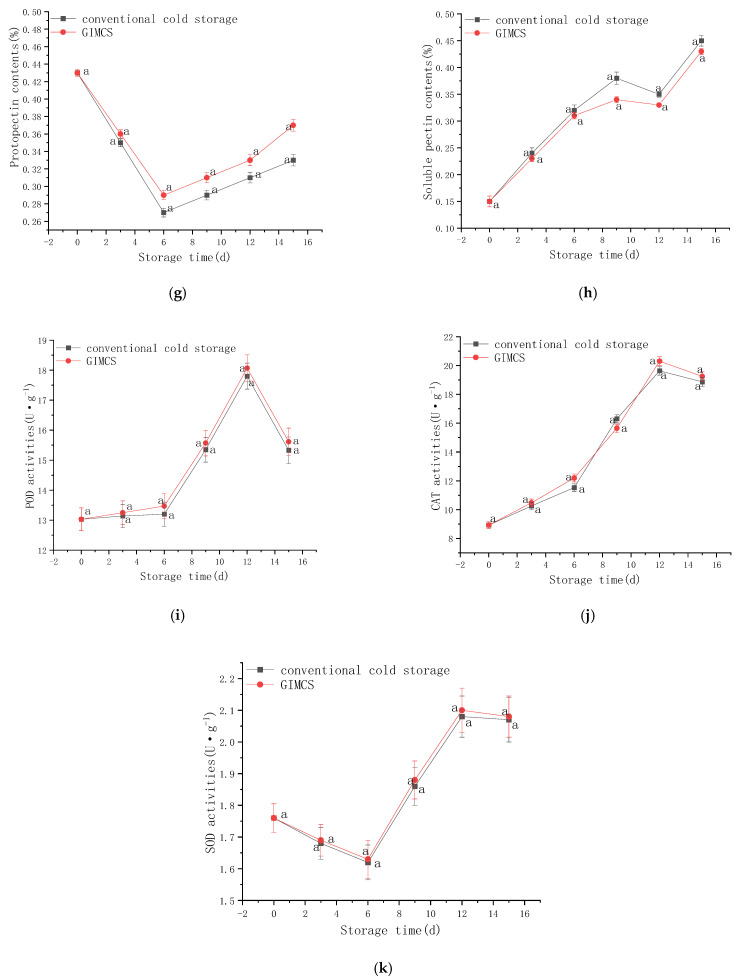
Effects of different storage environments on the fresh-keeping performance of cherry tomatoes, including (**a**) respiratory intensity, (**b**) weight loss, (**c**) hardness, (**d**) total soluble solid (TSS) content, (**e**) cellulase activity, (**f**) pectin esterase activity, (**g**) protopectin content, (**h**) soluble pectin content, (**i**) POD activity, (**j**) CAT activity, and (**k**) SOD activity. The same letters indicate that there is no significant difference in data among different treatment groups.

Respiration intensity is a direct indicator of fruit metabolic activity. As shown in Figure 17a, both the GIMCS and conventional cold storage groups exhibited a rapid increase in respiration rate during the early storage stage, reaching a peak on day 3, followed by a sharp decline. The peak respiration intensities were 603.88 mg CO_2_/(h·kg) for GIMCS and 604.58 mg CO_2_/(h·kg) for conventional cold storage, indicating nearly identical respiratory responses. By the end of the storage period, respiration intensities decreased to 375.34 mg CO_2_/(h·kg) and 376.35 mg CO_2_/(h·kg), respectively, with no statistically significant difference between the two groups (*p* > 0.05). These results demonstrate that GIMCS does not alter the intrinsic metabolic behavior of cherry tomatoes and that both storage methods exert comparable inhibitory effects on respiratory activity, effectively delaying metabolic senescence.

As illustrated in Figure 17b, the weight loss rate of cherry tomatoes increased progressively under both storage conditions. At the end of storage, weight loss reached 2.15% in conventional cold storage and 2.08% in GIMCS. Although the difference was not statistically significant, GIMCS consistently exhibited a slightly lower weight loss rate, indicating a tendency toward improved moisture retention. Importantly, weight loss remained below the 2.5% threshold in both systems, satisfying the requirements for short- to medium-term storage. The reduced dehydration observed under GIMCS conditions may help mitigate quality deterioration phenomena such as wilting and shriveling over extended storage durations, thereby better preserving visual appearance and textural integrity.

Firmness is a critical indicator of fruit texture and commercial quality, with its decline closely associated with softening processes. As shown in Figure 17c, firmness decreased steadily in both storage groups throughout the storage period, reflecting normal postharvest softening. No statistically significant differences were detected between the two treatments at any time point. Final firmness values were 1.26 kg·cm^−2^ for conventional cold storage and 1.30 kg·cm^−2^ for GIMCS, confirming comparable effectiveness in maintaining fruit texture. These results indicate that both storage systems effectively retard softening, thereby reducing susceptibility to mechanical damage and maintaining handling and market quality.

Total soluble solid (TSS) content is a key determinant of fruit sweetness and flavor. As shown in Figure 17d, TSS values in both groups exhibited a slight initial increase, followed by a gradual decline over the storage period. This trend is likely related to the incomplete physiological maturity of the fruits at harvest and transient metabolic activity during early storage. Although the GIMCS group consistently maintained marginally higher TSS levels than the conventional cold storage group, the differences were not statistically significant. By the end of storage, TSS values reached 5.22% and 5.28%, respectively. This consistent trend suggests that GIMCS may offer a modest advantage in preserving soluble solids and flavor attributes during later storage stages.

Cell wall-degrading enzymes play a central role in fruit ripening and textural degradation. Cellulase activity (Cx), which contributes to the breakdown of cellulose and cell wall structure [63,64], showed similar temporal trends in both storage groups, as illustrated in Figure 17e. Cx activity increased initially and peaked on day 6, reaching 66.31 mg·h^−1^·g^−1^ in conventional cold storage and 63.98 mg·h^−1^·g^−1^ in GIMCS, before declining thereafter. No statistically significant differences were observed between treatments throughout storage. Pectin methylesterase (PME), a key enzyme involved in pectin modification and cell wall disassembly [65], exhibited a continuous decrease in activity in both groups during storage (Figure 17f). Although PME activity was consistently slightly lower in the GIMCS group, final values of 21.78 mg·h^−1^·g^−1^ (GIMCS) and 22.22 mg·h^−1^·g^−1^ (conventional cold storage) did not differ significantly.

Overall, although the activities of cellulase and PME under GIMCS conditions were not statistically different from those observed in conventional cold storage, both enzymes consistently showed marginally lower activity levels throughout storage. This trend provides a plausible mechanistic explanation for the observed maintenance of firmness and soluble solids, suggesting that GIMCS may subtly suppress cell wall degradation and delay ripening-related physiological processes. Collectively, these results indicate that GIMCS offers preservation performance comparable to conventional cold storage, with potential incremental advantages in maintaining fruit quality and extending shelf-life stability.

Protopectin is a critical structural component and intercellular adhesive within the plant cell wall, and its enzymatic conversion to soluble pectin drives cell wall disassembly and tissue softening during postharvest ripening [66]. As shown in Figure 17g, protopectin content in cherry tomatoes decreased during the early storage period, reaching a minimum on day 6 in both the GIMCS and conventional cold storage groups, followed by a partial recovery. Notably, the GIMCS-treated fruits consistently retained higher protopectin levels throughout storage, suggesting a potential attenuation of cell wall degradation, although the difference was not statistically significant. Soluble pectin content (Figure 17h) progressively increased in both groups, reflecting ongoing pectinolytic activity. However, the GIMCS group exhibited a more gradual increase in soluble pectin accumulation, indicating a slower rate of protopectin breakdown. While the day 9 difference did not reach statistical significance, the consistent trend across multiple time points underscores its biological relevance. These observations suggest that GIMCS may better preserve cell wall integrity by modulating pectin metabolism, thereby contributing to improved textural stability during storage.

Protopectin retention is directly associated with firmness, whereas soluble pectin accumulation reflects enzymatic degradation of cell walls during ripening. The GIMCS group maintained higher protopectin levels and lower soluble pectin accumulation than the conventional cold storage group, providing mechanistic evidence for delayed softening. Although firmness measurements did not show statistically significant differences, the directional trends in pectin transformation support a biologically meaningful effect. These results indicate that GIMCS effectively modulates key aspects of cell wall metabolism, offering a promising strategy to enhance textural stability and prolong shelf life under cold storage conditions.

Plants rely on a coordinated antioxidant defense system to mitigate oxidative stress, primarily through the activity of superoxide dismutase (SOD), catalase (CAT), and peroxidase (POD) [67]. The temporal dynamics of these enzymes in cherry tomatoes stored under different conditions are shown in Figure 17i–k. POD and CAT activities initially increased, peaking on day 12, before gradually declining, reflecting a delayed but sustained response to postharvest oxidative challenges. SOD activity exhibited a triphasic pattern: initial suppression, followed by recovery, and subsequent decline, with a minimum on day 6 and a peak on day 12. While the overall activity trends were similar between treatments, GIMCS fruits consistently maintained higher POD and CAT levels at multiple time points, suggesting enhanced enzymatic resilience, although differences were not statistically significant. These findings indicate that both storage systems modulate antioxidant defenses in a temporally regulated manner, with GIMCS showing a trend toward improved maintenance of redox homeostasis, potentially supporting better postharvest quality.

The antioxidant enzyme system is crucial for delaying senescence and mitigating oxidative damage. The similar temporal activity patterns and magnitudes between GIMCS and conventional cold storage demonstrate that both treatments effectively maintain antioxidant capacity, thereby suppressing oxidative deterioration—including color darkening and nutrient loss—and contributing to an extended postharvest shelf life.

These results align closely with observations reported by Bremenkamp et al. [68], reinforcing the reliability of the findings. The superior insulation properties of GIMCS consistently maintained more stable internal temperatures, reducing metabolic deterioration compared with conventional cold storage, particularly under high-humidity conditions during the rainy season that can compromise conventional systems. Considering practical factors such as economic feasibility, operational convenience, and scalability, GIMCS represents a technically viable and sustainable alternative for postharvest storage, warranting broader adoption in horticultural supply chains.

## 4. Conclusions

This study addresses a critical gap in postharvest infrastructure for perishable agricultural products: the lack of scalable, cost-effective, and rapidly deployable cold storage solutions at production sites. Conventional cold storages are often constrained by large spatial requirements, labor-intensive assembly and disassembly, and limited portability, leading to substantial postharvest losses—particularly in perishable crops such as cherry tomatoes. To overcome these limitations, a novel temporary modular cold storage system, termed gas-inflated membrane cold storage (GIMCS), was developed. The findings demonstrate that GIMCS achieves performance comparable to conventional cold storages while offering substantial advantages in economic efficiency and operational flexibility.

1. Optimal Envelope Material and Insulating Gas

A single gas-inflated membrane composed of PA/PE (35%/65%) was identified as the optimal envelope material. The membrane exhibits a thickness of 70 μm and a gas column width of 2 cm, combining excellent mechanical performance with effective thermal insulation. Specifically, the longitudinal tensile strength reached 35.73 ± 0.03 N/mm^2^ and the transverse tensile strength reached 34.92 ± 0.02 N/mm^2^. The membrane also demonstrated superior pressure resistance and heat-sealing capability. Gas columns were filled with 100% CO_2_, achieving maximal thermal insulation by significantly reducing convective heat transfer.

2. Optimal Structural Configuration

A “double-insulation” configuration incorporating CF-GIMCS with dual thermal barriers—internal and external—was validated using a four-layer structure: “gas-inflated membrane + cotton + gas-inflated membrane + gas-inflated membrane”. This configuration delivers insulation efficiency equivalent to conventional cold storages and resolves the key limitation observed in earlier prototypes, where increasing layer count inadvertently degraded thermal performance due to enhanced solid-to-solid thermal conduction between layers.

3. Comparative Performance Results

Comparative testing between GIMCS and conventional cold storages of similar volumes (~50 m^3^) revealed that GIMCS reduced construction costs to 38% of those of conventional systems. Temperature distribution within GIMCS was highly uniform, with a maximum horizontal temperature differential of only 1.4 °C, comparable to conventional systems. No statistically significant differences were observed in core preservation metrics, including weight loss rate and respiration intensity. Notably, GIMCS demonstrated slightly better maintenance of fruit firmness (1.30 kg·cm^−2^) compared to conventional storage (1.26 kg·cm^−2^), indicating enhanced quality retention during storage. These results confirm that GIMCS meets the functional requirements for medium- to short-term on-farm cherry tomato storage.

4. Additional Advantages and Applicability

Beyond performance equivalence, GIMCS provides transformative logistical advantages over conventional cold storage, including rapid deployment, easy dismantling, mobility, self-supporting architecture, scalable volume adjustment, and high reusability. These features make it particularly well-suited for decentralized agricultural systems, such as small-scale and fragmented farming operations, enhancing the practicality of origin-level postharvest solutions.

5. Limitations and Future Work

This study focused exclusively on cherry tomatoes. Future work should extend the evaluation to other horticultural commodities and systematically investigate the long-term durability of GIMCS, including wind load resistance, structural aging, anti-puncture resilience, and operational stability under extreme climatic conditions. Collectively, these investigations will further validate GIMCS as a scalable, origin-level postharvest solution capable of reducing food loss in resource-constrained agricultural settings.

## Figures and Tables

**Table 1:** Tensile strength of gas-inflated membranes with different thicknesses.

	Thickness (μm)	30	50	70	90
Strength (N/mm^2^)					
Longitudinal		26.35 ± 0.02 d	31.50 ± 0.03 c	35.73 ± 0.03 a	33.24 ± 0.02 b
Transverse		26.09 ± 0.03 d	30.86 ± 0.02 b	34.92 ± 0.02 a	30.45 ± 0.02 c

Note: Different lowercase letters in the same column indicate significant differences (*p* < 0.05).

**Table 2:** Burst pressure characteristics of gas-inflated membranes with different thicknesses.

Thickness of Gas Column (μm)	30	50	70	90
Deformation of gas column (kPa)	80.3 ± 0.4 c	90.5 ± 0.5 b	108.0 ± 3.3 a	108.5 ± 3.4 a
Burst pressure of gas column (kPa)	85.5 ± 4.1 c	99.0 ± 2.2 b	110.6 ± 2.5 a	111.0 ± 3.7 a

Note: Different lowercase letters in the same column indicate significant differences (*p* < 0.05).

**Table 3:** Heat-sealing strength of membranes with different PA/PE ratios.

PA/PE	A (N)	B (N)	C (N)
25%/75%	22.60	19.04	18.86
30%/70%	27.93	26.50	26.44
35%/65%	35.90	32.08	31.25

PA/PE: Polyamide/Polyethylene.

**Table 4:** Reference data for gas thermal properties, safety limits, and market prices.

Gas	Thermal Conductivity (W/(m·K))	Safe Concentration	Price (USD/kg)
Hydrogen	0.1630	≤4% or ≥75.6%	9.74
Helium	0.1440	80%	6.96
Argon	0.0173	50%	1.11
Neon	0.0455	≤80%	12.53
Breath	0.0240	19.5–23.5%	0.70
Nitrogen	0.0224	≤88%	0.70
Carbon monoxide	0.0226	33.4	0.90
Carbon dioxide	0.0137	≤2%	1.22
Methane	0.3000	≤12%	1.67
Ethane	0.0180	≤5%	1.85
Propane	0.0148	≤2.1% or ≥9.5%	1.32
Ethene	0.0164	≤2.7% or ≥36%	0.49

USD: United States dollar.

**Table 5:** Calculated relationship between gas composition and thermal conductivity of gas mixtures.

Gas	Thermal Conductivity (W/(m·K))	Molar Mass (g/mol)	Mole Fraction				
CO_2_	0.0137	44	60%	70%	80%	90%	100%
Ar	0.0173	40	20%	15%	10%	5%	0%
N_2_	0.0224	28	20%	15%	10%	5%	0%
Thermal conductivity of mixed gas (W/(m·K))			0.01462	0.01438	0.0140	0.01392	0.01370

**Table 6:** Effect of gas column orientation optimization on thermal conductivity and thermal resistance.

Structure	Thickness (mm)	Thermal Conductivity (W/(m·K))	Thermal Resistance (m^2^·K/W)
Three layers of line and surface combination	38.7	0.115062	0.336341
Three layers of dots combined	41.0	0.126758	0.323452

**Table 7:** Cost comparison between GIMCS and conventional cold storage systems.

Cold Storage Type	GIMCS	Conventional Cold Storage
Gas-inflated membrane material price (USD/m^2^)	208.8	0
Other material price (USD/m^3^)	626.4	2750.04
Total price of thermal insulation materials (USD/each)	835.2	2750.04
Structural material price (USD/each)	320.16	278.4
Total cost of the cold storage wall (USD/each)	1155.36	3028.44

GIMCS: gas-inflated membrane cold storage; USD: United States dollar.

## Data Availability

The original contributions presented in this study are included in the article. Further inquiries can be directed to the corresponding author.
